# Hidden Comorbidities in Asthma: A Perspective for a Personalized Approach

**DOI:** 10.3390/jcm12062294

**Published:** 2023-03-15

**Authors:** Matteo Maule, Bianca Olivieri, Gabriella Guarnieri, Lucia De Franceschi, Nicola Martinelli, Rachele Vaia, Giuseppe Argentino, Andrea Vianello, Gianenrico Senna, Marco Caminati

**Affiliations:** 1 Allergy Unit and Asthma Center, Verona University Hospital, 37134 Verona, Italy; 2 Department of Cardiac Thoracic Vascular Sciences and Public Health, University of Padova, 35128 Padua, Italy; 3 Department of Medicine, University of Verona, 35134 Verona, Italy

**Keywords:** asthma, eosinophils, wheezing, α1-Antitrypsin deficiency, immunodeficiencies, sickle cell disease, cognitive impairment, obesity, cardiovascular risk

## Abstract

Bronchial asthma is the most frequent inflammatory non-communicable condition affecting the airways worldwide. It is commonly associated with concomitant conditions, which substantially contribute to its burden, whether they involve the lung or other districts. The present review aims at providing an overview of the recent acquisitions in terms of asthma concomitant systemic conditions, besides the commonly known respiratory comorbidities. The most recent research has highlighted a number of pathobiological interactions between asthma and other organs in the view of a shared immunological background underling different diseases. A bi-univocal relationship between asthma and common conditions, including cardiovascular, metabolic or neurodegenerative diseases, as well as rare disorders such as sickle cell disease, α1-Antitrypsin deficiency and immunologic conditions with hyper-eosinophilia, should be considered and explored, in terms of diagnostic work-up and long-term assessment of asthma patients. The relevance of that acquisition is of utmost importance in the management of asthma patients and paves the way to a new approach in the light of a personalized medicine perspective, besides targeted therapies.

## 1. Introduction

Bronchial asthma is the most frequent inflammatory non-communicable condition affecting the airways worldwide [1]. It is commonly associated with concomitant conditions, which substantially contribute to its burden, whether they involve the lung or other districts [1,2]. The most recent research has highlighted a number of pathobiological interactions between asthma and other organs in the view of a shared immunological background underling different diseases.

An extensive knowledge of asthma comorbidities, as well as the concomitant conditions potentially worsening the asthma course or, on the other hand, that are impacted by asthma in their evolution or burden, is essential for the specialist involved in their management. In fact, the awareness of those interactions should orient the diagnostic work-up, the follow-up schedule and the identification of a personalized pharmacological and non-pharmacological action plan. In that regard, an unexpected poor response to a targeted treatment, despite the eligibility criteria being matched, might suggest the need to perform a more extensive work-up aimed at identifying potential “hidden” comorbidities. According to the currently available data, the proportion of severe asthma patients not responding to biologics ranges approximately from 10 to 25% for anti-IL-5 [3,4,5] across different settings and specific monoclonal antibodies, which is not negligible in the light of a precision medicine approach. 

The present review aims at providing an overview of the recent acquisitions in terms of asthma concomitant systemic conditions, with a special focus on the pathobiological and immunological background linking different diseases, besides the commonly known asthma respiratory comorbidities.

## 2. Asthma in Eosinophilic Diseases

Bronchial asthma commonly expresses the respiratory tract involvement of eosinophil-related systemic conditions, including eosinophilic granulomatosis with polyangiitis (EGPA), allergic bronchopulmonary aspergillosis (ABPA) and hyper-eosinophilic syndrome (HES) [6]. Symptoms mimicking bronchial asthma might be also part of the clinical picture of other rare eosinophilic diseases, usually involving the upper and lower airways, e.g., chronic eosinophilic pneumonia (CEP), eosinophilic bronchitis (EB) and hyper-eosinophilic obliterative bronchiolitis (HOB) [7]. A proper diagnostic work-up of asthma patients, especially in the presence of elevated blood eosinophils and/or a difficult to control asthma, should always take into account the conditions mentioned above.

EGPA, previously known as Churg–Strauss syndrome, is a necrotizing granulomatous vasculitis affecting small to medium vessels [8]. It is a multisystem disorder characterized by asthma, chronic rhinosinusitis and eosinophilia but also lung, cutaneous, cardiovascular, neurological and gastrointestinal involvement. The presence of eosinophilia infiltrating the internal organs and the positivity of anti-neutrophil cytoplasmic antibodies (ANCAs) are typical of the disease [9]. Asthma is a central component of EGPA, representing the first of the three stages of the disease, which can overlap each other and progress at varying intervals. The first phase corresponds to asthma and allergic symptoms, followed by the phases of blood and tissue eosinophilia and finally necrotizing vasculitis [10]. Asthma is also part of the six diagnostic criteria for EGPA, established by the American College of Rheumatology (ACR) [11]. Severe or uncontrolled asthma is present in nearly 45% of patients at the time of diagnosis and is associated with a previous history of respiratory allergy and increased use of high-dose inhaled corticosteroids and oral corticosteroids for respiratory symptoms in the year prior to diagnosis of EGPA [12].

ABPA is a complex pulmonary disorder caused by a hyper-sensitivity reaction in response to colonization of the airways with Aspergillus fumigatus. It occurs almost exclusively in patients with asthma or cystic fibrosis, which are also the predisposing conditions necessary to diagnose ABPA. The main clinical features are asthmatic symptoms, mucus plugging, pulmonary opacities, bronchiectasis, lung fibrosis, elevated blood eosinophils count and total serum IgE, and detectable serum IgE or precipitating serum antibodies against Aspergillus fumigatus or aspergillus skin test positivity [13,14].

In CEP, a rare lung disease characterized by systemic and local eosinophilia with bilateral lung infiltrates, the most common symptoms are a prolonged cough and dyspnoea. Most patients have also an allergic disease, such as asthma, atopic dermatitis and allergic rhinitis. In addition to systemic corticosteroids, most patients use inhaled corticosteroids since they frequently have comorbid asthma [15].

HES is a group of rare disorders characterized by hyper-eosinophilia and organ damage and/or dysfunction attributable to eosinophils. Respiratory symptoms, such as a cough, dyspnoea and wheezing, are very common at presentation (25% of patients) and for this reason asthma could be frequently suspected [16]. Some patients are already diagnosed with asthma, while others are diagnosed after the diagnosis of HES [17]. Eosinophil-related manifestations usually are organ fibrosis, thromboembolism, cutaneous and mucosal signs and symptoms (erythema, angioedema, ulceration, pruritus and eczema) and peripheral or central neuropathy. Pulmonary involvement is common in HES and it is caused by eosinophilic infiltration of the lungs with subsequent fibrosis, heart failure or pulmonary emboli. The most common radiologic manifestations of pulmonary involvement are patchy ground-glass opacities and consolidation, but they can be various [18].

EB is an airway eosinophilic inflammation characterized by eosinophilia in induced sputum, bronchoalveolar lavage (BAL) and endobronchial biopsies, in the absence of bronchial hyper-responsiveness or airflow obstruction. The main symptom is a chronic non-productive cough that responds well to inhaled corticosteroids (ICS) treatment [19]. For these characteristics, EB can mimic asthma. In addition, some EB patients may develop chronic bronchial obstruction over time that would lead to the diagnosis of asthma [20,21].

HOB is a rare syndrome which causes chronic irreversible airflow obstruction that is unresponsive to bronchodilators or ICS. It is characterized by peripheral blood and/or BAL eosinophilia and radiological or histological evidence of bronchiolitis. This rare disease can mimic a form of severe asthma, but in some cases there appears to be an overlap with asthma, HES and other pulmonary eosinophilic diseases [19,22].

## 3. Alpha1-Antitrypsin Deficiency

α1-Antitrypsin deficiency (AATD) is a genetically determined condition that can affect several organs, leading to various clinical manifestations, including pulmonary emphysema, liver cirrhosis, vasculitis and panniculitis, which is an inflammatory skin disease. The most common clinically relevant AATD alleles are Pi*Z (Glu342Lys) and Pi*S (Glu264Val) [23].

AATD occurs in approximately 1:3000–5000 people; however, it continues to be underdiagnosed in patients presenting airflow obstruction [24,25]. In the European population, the frequency of homozygous AATD is estimated at 0.01–0.02% [26,27]. Data show that less than 10% of individuals with AATD are identified and that the delay is more than 5 years between initial symptoms and diagnosis [28].

The usual description of a patient with AATD as a young person with an unremarkable smoking history and end-stage emphysema is actually an uncommon presentation [29]. In a study in which 965 phenotypic tests were performed in patients with airway obstruction, the mean age of subjects with the Pi*Z genotype was 55.9 ± 9.8 years. In another study, the average smoking history of patients with ZZ at the time of diagnosis was 23.2 ± 14.5 pack-years [30].

AATD is frequently found in patients with asthma and, conversely, patients with AATD are more susceptible to develop asthma due to increased underlying lung inflammation, which leads asthma to coexist with AATD and emphysema [31]. Furthermore, it has been recognized that allergy and asthma often coexist with AATD (Table 1). In a study performed on patients with poorly controlled asthma, AATD was detectable in 2% to 3% of subjects, with 10.5% presenting a deficiency gene [32]. An Italian study reported a non-negligible prevalence of AATD in severe asthma patients on biologic treatment; a significantly more severe lung function decline was observed in non-smoking AATD patients, suggesting AATD as a potential cause of difficult-to-control asthma in this subpopulation [33]. A Spanish study that assessed the AAT distribution in an allergic asthma population reported that 22.4% of asthmatic patients had at least one mutated allele (S or Z) [34]. However, no association has been found between deficient AAT genotypes or serum AATD and development of severe asthma. Moreover, no correlation between ATT levels and FEV1 was observed. Another study highlighted that AAT heterozygosity does not appear to be an important risk factor for persistent airflow limitation in patients with asthma [35].

The symptoms and signs of AATD can be similar to the features of asthma, as more than 40% of patients have chronic sputum expectoration, even if they are non-smokers [36]. AATD itself may predispose to airway hyper-responsiveness, but the underlying biological mechanism has not yet been clarified [37]. Airway inflammation and remodeling in asthma involve degradation of the extracellular matrix, especially elastin, and are characterized by an imbalance between elastase and its primary inhibitor AAT [38]. As part of the normal physiologic response to infection and inflammation, neutrophil elastase (NE) degrades extracellular matrix components in the clearance of damaged tissue and may have other antibacterial and proinflammatory effects. In healthy individuals, AAT protects alveoli from the proteolytic effects of NE by maintaining a balanced environment between anti- and pro-inflammatory proteins in the lower respiratory tract. There are several lines of evidence implicating AAT as a participant in the immune response. In addition to NE, AAT is an acute-phase reactant. Upregulation of AAT occurs in response to infection and tissue injury, in order to promote tissue repair, in a mechanism driven by interleukin-6 (IL-6) and tumor necrosis factor (TNF) [29]. It has long been documented that NE is significantly increased in the induced sputum of asthma patients compared with control subjects, and NE levels in asthmatic patients correlate with a decline in FEV1 [39]. However, although fixed or non-reversible obstruction in patients with asthmatic symptoms in most cases is secondary to remodeling, in some cases it may be due to the underlying AATD. In these cases, the presence of symptoms, their onset in the fourth decade, a non-reversible airway obstruction and pan-lobular (pan-acinar) emphysema should raise suspicion and promote further investigations for AATD [40].

Patients diagnosed with AATD who have coexisting asthma need treatments aimed at reducing bronchial hyper-responsiveness and inflammation. Pneumococcal and influenza vaccinations significantly reduce the frequency of exacerbations in patients with AATD. Moreover, annual lung function assessments, pulmonary rehabilitation, liver disease screening, improving nutrition and the use of supplemental oxygen are important management tools. Clinical evidence has indicated positive effects on lung physiology with augmentation therapy, which can stabilize lung function and slow further lung destruction [29,41].

**Table 1:** Main distinguishing features of asthma and α1-Antitrypsin deficiency (AATD).

	Asthma	AATD
**Prevalence**	3–9% [42]	2–3% [33,38]
**Onset**	Variable, childhood > adulthood [42,43]	40–53 ys, Adulthood > childhood [38]
**Family history**	Allergic diseases (atopic eczema, allergic rhinitis, asthma, food allergies) [42]	Asthma, emphysema, liver cirrhosis, vasculitis [40,41]
**Lung function test (PFTs)**	Airway hyper-responsiveness, reversible obstruction [42]	Fixed or nonreversible obstruction [40,41]
**Concomitant allergies**	70% [42]	22% [43]
**Diagnosis**	Clinical history, physical examination, PFTs, demonstration of reversible obstruction [42,43]	Genetic testing, and phenotype testing [38,43]
**Therapy**	ICS, LABA, LAMA, montelukast, Biologics [42,43]	As asthma + AAT augmentation therapy [38,43]

## 4. Immunodeficiencies

Immunodeficiencies are conditions characterized by a defective immune system functioning which leads to increased susceptibility to infections, cancer and autoimmunity. Common Variable Immunodeficiency (CVID) is the prototype of these conditions being the most common symptomatic primary immunodeficiency (PID).

The presence of an immune deficiency in an asthma patient increases the burden of the disease, leading to poorer symptom control, higher exacerbation rates and impaired lung function over time [44].

Asthmatic patients have an increased risk (OR 2.46–4.98) of having an antibody deficiency compared to those without a history of asthma [45] and in CVID patients, asthma is the most common respiratory complication [46]. The reason underneath this association is still poorly understood, although there is probably a role played by corticosteroid therapy, a well-known cause of secondary immunodeficiency [47].

The coexistence of asthma and immunodeficiency determines a worse prognosis, as demonstrated by a recent study which showed that asthmatic patients with PIDs more frequently have a severe condition and frequent exacerbations [48].

A compromised immune system leads to an increase in respiratory infections, which in turn are triggers for asthma exacerbation [49]. Therefore, if frequent exacerbations are present, the clinician must pay particular attention and request additional tests (i.e., Ig dosage and test vaccine response) in order to identify a possible underlying immunodeficiency. Another subgroup of particular interest is non-type two asthma with low serum eosinophils and fractional exhaled nitric oxide (FeNO), as these patients more frequently present an antibody deficiency [50].

In asthmatic patients with immunodeficiency, the use of immunoglobulin replacement therapy (IRT) or prophylactic antibiotic therapy has been proposed with good results [44,49]. In a recent study, Tiotiu and colleagues showed that IRT and prophylactic azithromycin were able to reduce the number of infection-driven exacerbations, increase asthma control and improve lung function. Moreover, patients who do not respond to azithromycin may benefit from IRT, opening up the possibility of a sequential approach [44]. IRT has also been studied in patients with severe asthma without an antibody deficiency, but failed to show a significative improvement, highlighting the importance of careful patient selection alongside consideration of the high cost [51].

To date, it is not completely clear whether the primum movens is asthma or immunodeficiency; probably, both conditions represent a fertile ground where the other can develop and they act synergically in worsening the patient’s condition. The possible presence of an immunodeficiency in asthmatic patients is an issue that must be addressed, especially in the presence of certain features/red flags, in order to increase disease control, improve quality of life and avoid complications.

## 5. Sickle Cell Disease

Sickle cell disease (SCD) is the most common worldwide distributed monogenic red cell disorder, which still has high mortality and morbidity. The main clinical manifestations of SCD are chronic hemolytic anemia and acute vaso-occlusive crisis (VOCs) [52,53]. These are the major causes of hospitalization in both pediatric and adult patients with SCD. Recent progresses in pathophysiologic understanding of SCD have highlighted the key role of sustained oxidation and chronic inflammation associated with increased pro-inflammatory cytokines (e.g., IL-1b, IL-6 and endothelin-1 (ET-1)) in the generation of VOCs. In addition, the unbalance between pro-inflammatory and pro-resolving mechanisms promotes inflammatory vasculopathy and disease progression [54,55]. The biocomplexity of SCD is further increased by the local relative reduction in nitric oxide bioavailability, contributing to abnormal vascular tone and to a plasma pro-oxidant environment [55,56,57]. Lungs are one of the target organs of SCD due to the peculiarity of the vascular bed where deoxygenated sickle red cells transit [58]. In addition, non-genetic modifiers targeting lung have been shown to contribute to sickle cell related pulmonary manifestations such as air quality, weather (e.g., humidity, wind speed or temperature), smoking or infections [59].

A broad spectrum of lung clinical manifestations, such as acute chest syndrome (ACS), asthma and recurrent wheezing, venous thromboembolic disease and sleep-disordered breathing, commonly coexist with SCD [60]. The association between asthma and SCD has long since been recognized; however, the frequent wheezing that characterizes SCD patients may perhaps account for overdiagnosis of asthma in such a condition [61]. Of note is that the presence of wheezing, even in the absence of an asthma diagnosis, has been recently proposed as a risk factor for the severity of sickle cell related clinical manifestations [62]. This is of interest since children with SCD and asthma have increased rates of VOCs and ACS and have an increased risk of premature death [61,63]. Epidemiologic studies report a higher prevalence of asthma in the SCD population compared to matched healthy controls. However, the small group size, patients’ heterogeneity and the lack of a standardized diagnostic approach are the major limitations of these studies. These factors justify the presence of few contradictory results on the prevalence of asthma in patients with SCD [64,65]. In this scenario, the design of population-based studies comparing SCD patients and healthy control subjects in terms of asthma incidence and prevalence are required to drive consistent conclusions on asthma in SCD, particularly considering that recurrent wheezing is a common feature in patients with SCD, even without a personal or familiar asthma history. In a study on 939 patients experiencing ACS, 11% of them presented with wheezing on admission to the hospital [61]. Similar findings have been confirmed by different research groups, describing an equally common wheezing presentation of ACS among children and adults [66,67]. Notably, severe wheezing, regardless of the presence of underlying asthma, was reported to double the rates of pain and ACS, decrease lung function and increase risk of death [68,69].

Different factors have been suggested to contribute to airways obstruction in SCD, such as increased Th2 response and pulmonary vascular engorgement due to the presence of dense, sticky red cells, overactivated neutrophils and inflammatory vasculopathy [70]. This mediates abnormal gas exchange leading to bronchoconstriction-induced ventilation–perfusion mismatch and tissue hypoxia associated with increased small airway “compression” and resistance. The presence of high pulmonary ET-1 expression combined with reduced NO bioavailability and autonomic dysfunction has been proposed to play a key role in increased airway hyper-reactivity observed in patients with SCD [71]. Some of these features are shared by asthma as well as endothelial disfunction, which is also a key element in the pathogenesis of obstructive pulmonary diseases [72].

Asthma impacts on SCD patients differently from the general population; thus, the investigation of signs and symptoms of asthma (or asthma-like diseases) in SCD should be part of the comprehensive care of subjects with SCD, in particular for children and adolescent populations [62,70]. This will allow us to early intercept patients at risk of developing a severe clinical phenotype. Whenever a diagnosis of asthma is confirmed, clinical management should be based on international recommendations and guidelines, including inhaled corticosteroids and bronchodilator treatments according to asthma severity grade. The use of systemic corticosteroids should be carefully evaluated as they are associated with an increased risk of rebounding SCD-related pain on withdrawal and avascular necrosis especially in SCD patients [60,73,74]. New biologic therapies might be considered and discussed case by case in a multidisciplinary team, as they could allow for better asthma control and lower steroid use.

## 6. Neurodegenerative Diseases

In recent years, several studies revealed an intriguing relationship between asthma and brain involvement [75,76,77,78,79,80,81]. Mild cognitive impairment (MCI) was the most frequently reported disease, though dementia (D) and Alzheimer Disease (AD) were also associated with asthma, but to a lesser extent. In these epidemiological studies, older age, uncontrolled and more severe asthma were identified as risk factors for brain involvement, but several studies confirmed this association even at a younger age and in patients with mild asthma [82]. A bidirectional interaction between asthma and extra-respiratory systemic diseases has been recently demonstrated. In fact, in a subgroup of patients with more severe asthma, a significant relationship with concomitant hyper-tension and metabolic syndrome has been revealed, the increase in interleukin-6 (IL-6) being the hallmark of this group of patients [83]. Bronchial asthma can negatively affect metabolic diseases, such as diabetes or vascular diseases, even at a early age [84,85]. The drop in oxygen saturation, which can complicate asthma exacerbations, has been indicated as being responsible for neural involvement. Neural changes due to hypoxia might translate into neuronal damage, particularly in regions of high metabolic demand [82]. However, MCI has also been found in mild asthma and no correlation has been proved between the frequency of exacerbations and neural involvement. More recently, asthma-associated inflammation has also been recognized as responsible for inflammation of the central nervous system leading to glial activation and eventually cognitive impairment. A recent study identified a significant increase in astrocytes activation markers, as GFAP (Glial Fibrillary Acid protein) and NfL (Neurofilament Light Chain), which was significantly related to asthma severity. In the same study on asthmatics, differences have been found in several metrics of diffusion-weighted magnetic resonance imaging (MRI), suggestive of neuroinflammation and neurodegeneration, but no correlation has been found between T2 and neural inflammation biomarkers [86]. However, concomitant treatment with biologics or steroids may have influenced this result. Furthermore, using functional MRI after a specific allergen challenge, asthmatic patients who presented a late response displayed a different activation of the anterior insular cortex in comparison to healthy controls and patients with mild asthma, suggesting the existence of different neurophenotypes in asthma [87]. Likewise, in younger asthmatics a significant reduction of the metabolites, such as N-acetyl aspartate, glutamate, creatine and myo-inositol, from hippocampus was detected, indicating neural viability, reduced energy reserve and glial activation. It has been hypothesized that this alteration might precede the subsequent MCI at an older age [88]. In the relationship between asthma and the central nervous system, the role of the treatment must be considered. One study showed that a prolonged use of oral corticosteroids reduced the volume of the amygdala, which is a brain structure involved in the emotional pathway and plays an important role in the response to stress [89]. On the other hand, inhaled steroids proved to have a protective role in animal models as well as in humans whereas the exclusive use of bronchodilators has a negative impact on brain involvement, probably due to the risk of uncontrolled asthma [82]. In addition, montelukast has been found to have a protective role against damage to the nervous system in animal models [90]. Though the association between asthma and nervous system involvement must be confirmed by further studies and its mechanisms clarified, current data might have potential social implication. MCI, dementia and AD are common diseases in older age and, notably, no treatment is currently available. They can negatively also affect the management of concomitant diseases, strongly reducing the adherence to their treatment. Therefore, a prompt identification of an initial brain involvement could be vital in term of prevention and progression of these diseases.

## 7. Metabolic Syndrome and Obesity

Metabolic syndrome consists of a cluster of risk factors that increase the chance of developing heart disease, strokes and type 2 diabetes. These conditions include increased blood pressure, high blood glucose, excess body fat around the waist and abnormal HDL cholesterol or triglyceride levels. The prevalence of metabolic syndrome among patients with asthma is estimated to be 25% [91]. A recent study has shown that metabolic syndrome is more prevalent in asthma patients compared to controls, and that the onset of asthma in adulthood is associated with metabolic syndrome, even when adjusted for age, sex, body mass index and smoking history [92]. Among the components of metabolic syndrome, two are mainly associated with an increased risk of incident asthma in adults: high waist circumference and elevated glucose or diabetes.

According to the HUNT study, a prospective cohort study, patients with hyper-glycaemia or type two diabetes mellitus have a higher incidence of asthma (OR 1.43, 95% CI 1.01–2.04) over an average of 11 years of follow-up [93]. Higher glycated haemoglobin (HbA1c) is associated with a higher asthma exacerbation rate compared with individuals with normal HbA1c: patients in the prediabetes range have a 27% higher rate and those in the diabetes range have a 33% higher rate [94]. In addition, HbA1c level in the pre-diabetes/diabetes range is associated with a higher rate of asthma hospitalization and inversely related to respiratory function parameters, such as FEV1 and FVC [95]. Among the mechanisms underlying the association between asthma and diabetes, insulin resistance appears to play an important role leading to airway disfunction [96]. In fact, hyper-insulinemia associated with insulin resistance is associated with the proliferation of airway smooth muscle and promotes its contractility [97].

Hyper-tension is associated with worse morbidity and outcomes in asthma, although there are few studies supporting this association [2]. A large study reported that hyper-tension correlated with greater odds of rescue inhaler use, emergency department visits or hospitalizations, and corticosteroid use [98]. Hyper-tension and asthma share some immune mechanisms in their pathogenesis, especially for non-type two asthma. Angiotensin II is a vasopressor that induces secretion of IL-17A, which blocks vasodilation induced by endothelial-derived nitric oxide [2]. IL-17A seems to play a key role because IL-17A knock-out mice do not experience prolonged hyper-tension [99], and, on the other hand, this cytokine contributes to airway hyper-responsiveness in mice [100] and is elevated in bronchoalveolar lavage fluid of severe asthma patients [101]. For this reason, angiotensin receptor blockers have been evaluated as a dual treatment for both asthma and hyper-tension [102].

Regarding elevated blood lipid levels, several studies have been conducted on adult patients with asthma with conflicting results [103,104,105]. A large meta-analysis revealed that levels of low-density lipoprotein (LDL) and total cholesterol were higher in patients with asthma than controls, but there was no association between asthma and levels of high-density lipoprotein (HDL) or triglycerides [106]. A recent study showed that triglycerides were statistically significantly higher in patients with obesity and asthma adjusted for body mass index (BMI), blood eosinophils and statin use [107]. In addition to regulation of cholesterol metabolism, the anti-inflammatory properties of statins are believed to play a role in asthma control [108,109]. In a recent case-control study, statin use was associated with a reduced risk of asthma-related emergency department visits, hospitalizations and systemic steroid use in patients with asthma [110].

There is a complex interrelationship that links asthma to obesity. Asthma prevalence among obese patients is increased compared to the general population and data show that 20–50% of severe asthmatic patients are obese, with female patients at higher risk. The reasons underlying such interplay are not fully understood but genetic, dietary, environmental and immune factors are thought to play a major role in the pathogenesis of both conditions with an emerging role of the gut microbiome [111].

Traditionally, asthmatic–obese patients were considered to have a specific disease pheno-endotype characterized by neutrophilic inflammation, lower allergic burden, less airway hyper-responsiveness and worse response to inhaled therapy. To date, the picture is more complex as various asthma phenotypes have been identified in the obese population, although usually with a consistent Th1 fingerprint [112].

What is ascertained is that the association between asthma and obesity determines worse symptom control, reduced response to inhaled therapy and higher exacerbation and hospitalization rates [111]. Different mechanisms play a role in the aggravation of the clinical condition in asthmatic–obese patients. First of all, the increased thoracic and abdominal adipose tissue reduces pulmonary expansion adding a restrictive component to lung functional impairment; secondly, the increased systemic inflammatory status that characterizes obese patients leads to higher airway obstruction and reduced response to corticosteroids [113]. Not only obesity itself, but also comorbidities typically associated with obesity are involved in the aggravation of asthma symptoms: obese patients are more likely to have metabolic syndrome and suffer from obstructive sleep apnoea syndrome, GERD and depression [42].

The approach to therapy is particularly delicate in obese–asthmatic patients, who are said to be less likely to be adequately controlled by standard inhaled therapy. The use of long-term systemic corticosteroids should be avoided given the already high burden of metabolic comorbidities in such patients. Concerns rise for biologic therapies as well, since absorption and distribution are probably altered and standard dosage could be insufficient to have a therapeutic effect in obese patients, as this happens in other diseases [114]. Moreover, it is possible that traditional biomarkers would not be appropriate in this population and lower thresholds, for example for eosinophils, have been proposed to identify specific inflammatory patterns [115].

In conclusion, asthma in obese patients should be considered as a different and more complex disease when compared to asthma in non-obese patients. It is characterized by respiratory and systemic additional cofactors and comorbidities that make both endotypization and phenotypization more complex and less efficacious, and therefore it needs an integrated diagnostic and therapeutic approach.

## 8. Cardiovascular Disease

Beyond their own phenotypic characteristics and differences, both asthma and atherosclerosis are chronic inflammatory diseases in which the pathways and the effectors of the inflammatory response play a crucial role. Atherosclerosis, the leading histopathologic ground of cardiovascular disease (CVD), is per se a low-grade chronic inflammatory condition and not only a lipoprotein-driven disease [116]. Lymphocytes, monocytes, macrophages and neutrophils are important participants at the various stages of CVD progression, from early development to late complications of atherosclerotic plaques [117,118]. Noteworthy from a therapeutic point of view is the importance of inflammation in CVD, which has been further emphasized by the proof-of-concept CANTOS (Canakinumab Anti-Inflammatory Thrombosis Outcomes Study) trial, which showed that anti-inflammatory treatments—in that case, the pharmacological inhibition of interleukin-1beta—may reduce cardiovascular risk independently of lipoprotein-mediated mechanisms [119]. However, the association between inflammation and CVD may go beyond the “classic” inflammatory pathways. Recent evidence addressed the interest in allergic inflammation and its cellular effectors, such as mast cells, eosinophils and basophils [120,121].

In mice models, several studies indicated that mast cells may promote atherosclerosis by releasing proinflammatory cytokines. Mast cell-deficient mice were protected from the development of atherosclerosis when crossbred with low-density lipoprotein receptor (LDLR)-deficient mice [122] and failed to develop abdominal aortic aneurysms induced by elastase perfusion or periaortic chemical injury [123]. On the other hand, the activation of mast cells in wild type mice resulted in enhanced abdominal aortic aneurysms growth, while mast cell stabilization with disodium cromoglycate diminished abdominal aortic aneurysms formation [123]. Pharmacological stabilization of mast cells by means of disodium cromoglycate attenuated experimental atherogenesis in LDLR-deficient mice [124], while the mast cells stabilizer cromolyn markedly decreased the rupture rate of aneurysms in adult mice with induced intracranial aneurysms [125]. According to this experimental evidence, pharmacological inhibition of mast cell activation has been hypothesized to offer new therapeutic strategies aiming to reduce cardiovascular risk in humans [120].

Eosinophil-deficient mice had also decreased atherosclerotic lesion burden as compared with littermate controls when crossbred with apolipoprotein E (Apo E)-deficient mice. Eosinophils have been shown to contribute to atherosclerotic lesion formation by activating endothelial cells, as well as to support arterial thrombosis by propagating platelet accumulation [126]. In humans, genome-wide association studies (GWAS) showed that sequence variants affecting eosinophil numbers were associated not only with asthma but also with myocardial infarction [127]. Eosinophil blood count has been proposed as a potential predictor of cardiovascular risk [128,129] and a recent large cohort study within the framework of the Rochester Epidemiology Project confirmed that high eosinophil blood counts were associated with an increased risk of cardiovascular events, including heart failure, stroke and cardiovascular death [130]. Moreover, eosinophil degranulation and activation have been linked with coronary instability and a worse clinical outcome after acute myocardial infarction [131].

Few biochemical and clinical clues have suggested a role for basophils in CVD so far. Nonetheless, basophil activation was higher in subjects with acute coronary syndrome than in those with stable angina [131]. Within the framework of the angiographically controlled Verona Heart Study cohort, basophil blood counts predicted mortality in patients with clinically stable coronary artery disease, with high cell counts associated with an increased risk of total and cardiovascular mortality. Additionally, high basophil blood counts were associated with enhanced factor II plasma coagulant activity, thereby suggesting an underlying prothrombotic diathesis [132]. Notably, basophils can release extracellular DNA traps facilitating coagulation activation and thrombus formation [133], while basophil granules contain polyphosphate, which is recognized as a procoagulant player in haemostasis [134,135].

Therefore, the cellular effectors of allergic inflammation, such as mast cells, eosinophils and basophiles, could be a potential pathogenic link between asthma and CVD.

In line with these observations and consistent with the potential role of allergic inflammation in CVD, several epidemiological studies showed an increase in cardiovascular risk in patients with asthma, although with some controversial results [136]. Asthma has been associated with endothelial dysfunction [137], prothrombotic diathesis [138], myocardial infarction [139] and cardiovascular mortality [140,141]. Asthma has been associated with atherosclerotic artery changes independent of traditional cardiovascular risk factors [84] and with ischemic heart disease independent of pharmacological therapies [142]. A very recent meta-analysis confirmed that asthma patients have an increased risk of CVD morbidity and mortality [143]. Allergic asthma aggravated atherosclerosis even in Apo E-deficient mice models, where a potential influence on cholesterol metabolism was also detected [144].

According to some evidence, asthma patients present an increased risk of right heart dysfunction as well. The first report dates back to early 20th century when right heart-related electrocardiographic alterations were described in acute bronchial asthma by different research groups [145]. Of note, such alterations resolved after the resolution of the asthmatic attack. The effects of asthma on right heart functioning were highlighted outside the acute attack as well, as demonstrated by a recent study on 55 patients with mild–moderate asthma which showed the presence of subclinical right heart impairment derived from abnormal echocardiographic parameters [146]. It is not surprising if we consider the vasoconstrictive response and remodeling of pulmonary arterioles secondary to hypoxia and followed by increased vascular resistance. It may lead to pulmonary hyper-tension and eventually upstream right heart disfunction due to the enhanced right ventricle afterload [147]. Such changes seem to correlate with asthma severity and develop early in the disease course; in fact, they can be detected in asthmatic children who have been shown to have increased right ventricle wall thickness [148] as well as significantly different right heart related echocardiographic parameters and NT-pro BNP blood levels, when compared to healthy controls [149].

Such findings highlight that asthma has a deep impact on right heart function as well, particularly on diastolic function, being responsible for both acute and chronic/progressive alterations which should be carefully assessed when evaluating patients. Echocardiography, when performed by trained clinicians able to appraise specific right heart-related parameters, is the most accessible screening tool, along with laboratory markers such as NT-proBNP, which improves the diagnostic accuracy. An early diagnosis, intercepting the subclinical phase when alterations are still entirely or partially reversible, should not be neglected in asthma patients of any age.

From a therapeutic perspective, when considering that inflammation is the most important underlying mechanism in asthma, anti-inflammatory therapies including steroids or biologicals seem to be relevant in preventing damage and disease progression, although data are still scarce [150].

In summary, taking into account all this clinical and experimental evidence, it is tempting to speculate that it may be time to rethink both the role of allergic inflammation in atherothrombosis and the link between asthma and CVD. From a pathobiological point of view, allergic inflammation pathways may be relevant in the development, progression and complications of atherosclerosis and in both left and right heart function impairment. Patients with asthma may have an increased cardiovascular risk and clinicians should be aware of this issue. Finally, in perspective, a more thorough knowledge of inflammatory pathways in CVD may both (i) pave the way to new therapies for reducing the residual cardiovascular risk and (ii) lead to more efficient treatment strategies for improving overall survival and quality of life in asthma patients.

## 9. Conclusions

**Figure 1:**
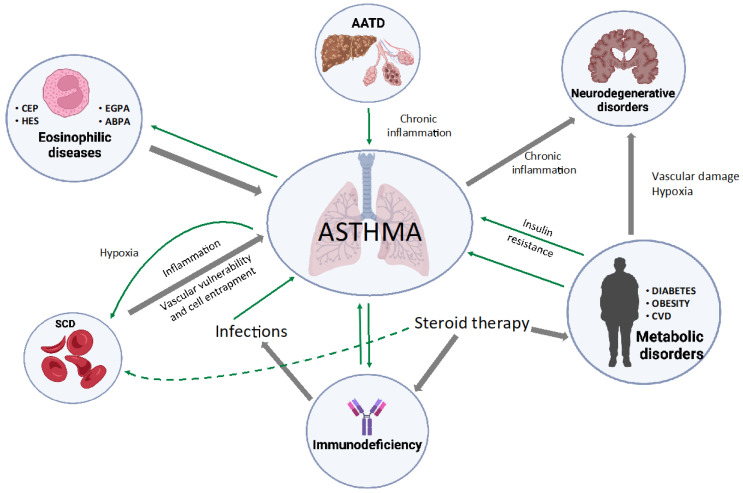
Overview of different conditions potentially coexisting and interacting with bronchial asthma. Grey, thick arrows indicate a pathobiological contribution in the development of the disease; green, thin arrows highlight a negative impact on the disease control, burden and evolution. AATD: α1-Antitrypsin deficiency; ABPA: allergic bronchopulmonary aspergillosis; EGPA: eosinophilic granulomatosis with polyangiitis; CEP: Chronic Eosinophilic Pneumonia; CVD: cardiovascular Disease; HES: Hyper-eosinophilic Syndrome; SCD: Sickle Cell Disease.

In the era of a personalized approach to patients, the availability of different precision medicine options is not enough. In fact, the effective use of targeted treatments does not allow for covering the complex interplay of concomitant conditions potentially coexisting in each patient. On the other hand, the recent insights coming from the translational and pharmacological research have revealed more than one pathobiological connection, linking different conditions and pleiotropic clinical manifestations (Figure 1). Under that perspective, every clinician managing asthma patients, or individuals affected by conditions which may hamper the achievement of asthma control or whose burden may be negatively impacted by coexisting asthma, should be aware of those interactions and tailor his or her clinical approach accordingly. In other terms, from the diagnostic work to the identification of the best combination of targeted treatments, ordinary therapy and a non-pharmacological approach as well as an extensive knowledge of the link between apparently different diseases and their common immunological background is essential.

The relevance of that acquisition is of the utmost importance in the management of asthma patients and paves the way to a new approach in the light of a personalized medicine perspective.

## Data Availability

Not applicable.
